# Towards a semi-automatic functional annotation tool based on decision-tree techniques

**DOI:** 10.1186/1753-6561-2-s4-s3

**Published:** 2008-12-17

**Authors:** Jérôme Azé, Lucie Gentils, Claire Toffano-Nioche, Valentin Loux, Jean-François Gibrat, Philippe Bessières, Céline Rouveirol, Anne Poupon, Christine Froidevaux

**Affiliations:** 1 LRI – CNRS UMR 8623 – University Paris-Sud 11, F-91405 Orsay Cedex, France; 2 INRA, Unité Mathématique, Informatique et Génome UR1077, F-78352 Jouy-en-Josas, France; 3 LIPN – UMR CNRS 7030 – Institut Galilée – University Paris-Nord, F-93430 Villetaneuse, France; 4 IBBMC – CNRS UMR 8619 – University Paris-Sud 11, F-91405 Orsay Cedex, France

## Abstract

**Background:**

Due to the continuous improvements of high throughput technologies and experimental procedures, the number of sequenced genomes is increasing exponentially. Ultimately, the task of annotating these data relies on the expertise of biologists. The necessity for annotation to be supervised by human experts is the rate limiting step of the data analysis. To face the deluge of new genomic data, the need for automating, as much as possible, the annotation process becomes critical.

**Results:**

We consider annotation of a protein with terms of the functional hierarchy that has been used to annotate *Bacillus subtilis *and propose a set of rules that predict classes in terms of elements of the functional hierarchy, i.e., a class is a node or a leaf of the hierarchy tree. The rules are obtained through two decision-trees techniques: first-order decision-trees and multilabel attribute-value decision-trees, by using as training data the proteins from two lactic bacteria: *Lactobacillus sakei *and *Lactobacillus bulgaricus*. We tested the two methods, first independently, then in a combined approach, and evaluated the obtained results using hierarchical evaluation measures. Results obtained for the two approaches on both genomes are comparable and show a good precision together with a high prediction rate. Using combined approaches increases the recall and the prediction rate.

**Conclusion:**

The combination of the two approaches is very encouraging and we will further refine these combinations in order to get rules even more useful for the annotators. This first study is a crucial step towards designing a semi-automatic functional annotation tool.

## Background

### Context

Due to the continuous improvements of high throughput technologies and experimental procedures, the number of sequenced genomes is increasing exponentially. The first sequenced genome was published 10 years ago. Currently, about 800 (updated in May 2008) genomes have been completely sequenced and published, coding for more than 6 millions proteins (as stored in the protein sequence database UniProtKB). A further 3 700 new genomes are expected in the near future [1].

Biologist experts play a central role in the analysis of this massive amount of raw data. To annotate a new genome they need to integrate many pieces of information coming from various sources: results of bioinformatics analysis programs, data stored in specialized databases, results of high-throughput experiments such as transcriptomics, proteomics, etc., information stored in the literature, general knowledge about the domain of interest (biological properties of the studied organism, its ecology, etc.). Even for a small bacterial genome, containing about 2 000 genes, this annotation task is a heavy burden that takes between 12 and 18 months to complete for a small team of annotators. A number of annotation tools have been designed to help the biologists concentrating exclusively on this high-level task. The aims of theses tools are to hide technical details, to make the system implementation transparent, to centralize and facilitate the access to relevant data, and to report a synthesis of all the findings to the annotators in an efficient manner. In spite of these tools, the need for a human supervision of the annotation process still constitutes the bottleneck of genomic data analyses. Therefore, to face the deluge of new genomic data, there is a crying need to automate, as far as possible, the annotation process itself. Computational annotation methods should take into account as much relevant information as possible regarding the analyzed genome, as human experts do.

Let us emphasize here that there is a difference between the direct annotation of the gene product, e.g., "fatty acid-binding protein, adipocyte" and the annotation of the protein with terms of a functional hierarchy, for instance for GO [2], "GO:16564; Molecular function: transcription repressor activity" or "GO:42632; Biological process: cholesterol homeostasis". In the latter case different proteins are grouped according to their molecular function or to the functional path they belong to. In this article we are concerned with the second type of annotation.

Annotation is mostly based on evolutionary considerations, more precisely on the concept of homology. Homology is the fact, for two genes or proteins, to descend from a common ancestor. As such they share a number of properties, in particular their function. The principle of annotation is thus to infer an homology relationship between a gene (protein) of interest and a gene (protein) whose function is known and to transfer this function.

### State of the art

Computational annotation methods range from symbolic to numerical techniques. Some of them are based on machine-learning techniques (e.g. SPEARMINT [3] or GOPET [4] that use C4.5 [5] and SVM [6] respectively) while others are probabilistic approaches (e.g. MAGIC [7,8] which is based on a Bayesian network or the Bayesian approach proposed in [9]).

In the context of the RAFALE project [10] our goal is to provide biologists with a *semi-automatic *tool for functional annotation. As a straightforward consequence, both productivity of the annotators and consistency of the annotations would be improved. It is a semi-automatic tool in the sense that the process is collaborative: annotations are suggested by rules that reflect known protein annotations but the annotations are ultimately validated by the biologists. We chose to learn rules obtained through decision-trees that exhibit several good features. They can be easily understood and used by human annotators. They represent modular pieces of information that can be considered as explanations of the annotations proposed to users. In our approach not only do we aim at obtaining good quality annotations but also we focus on *how they have been obtained*. This point is essential for a relevant evaluation of the quality of the annotations in order for them to be used by the biologists. Otherwise, biologists would not trust such rules and would not use them, thus missing a possibility of saving time. However, we do not restrict ourselves to high quality annotations. Unlike HAMAP [11], we can be led to propose several alternative annotations, together with their confidence degree, asking biologists to conclude themselves. In the following, we propose to apply two decision-trees techniques to the problem of predicting classes from a functional hierarchy, in the same spirit as in [12] which deals with the problem of predicting ORF functional classes. Two different frameworks have been chosen to represent rules that are more or less expressive and accordingly more or less expensive: first-order decision-trees [13] and multilabel attribute-value decision-trees [14]. As we are more interested in providing biologists with reliable annotation – even though it concerns only a restricted subset of proteins – we aim at obtaining rules with high precision rather than good recall (see section Results).

## Methods

### Annotation framework and genomes under study

#### The available data

In this work our training set corresponds to data provided by the AGMIAL annotation platform [15]. This platform has been used to annotate two lactic bacteria: *Lactobacillus sakei *[16] and *Lactobacillus bulgaricus *[17].

AGMIAL embodies an annotation strategy that considers the following pieces of information:

• modular aspect and intrinsic properties of protein sequences;

• search for homology relationship between proteins;

• genomic context;

• subcellular localization.

More than 30 bioinformatics methods belonging to the above categories are implemented in AGMIAL. As mentioned in the Background section, homology search techniques represent the cornerstone of the annotation process. However, with the availability of many sequenced genomes and thus the possibility of annotating a new genome in the light of other known genomes, techniques based on the genomic context are becoming increasingly important.

**Figure 1:**
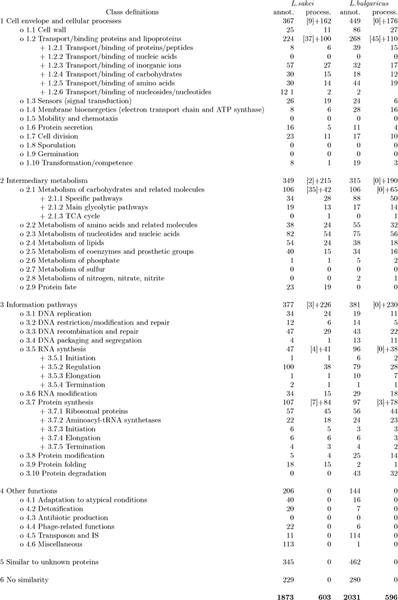
**Functional hierarchy used to annotate *B. subtilis***. The left hand side of the figure shows the three level functional hierarchy. The columns on the right hand side correspond to the number of proteins annotated with the corresponding node or leaf for *L. sakei *and *L. bulgaricus *respectively. Columns 'annot.' correspond to the number of proteins annotated by human experts, columns 'process.' correspond to the number of proteins that are considered in this study (see text). In the latter case, for inner nodes, this number is given as the sum of the figures for the direct descendants and the number of proteins having the node (partial) annotation, within square brackets. For instance, for proteins of *L. sakei *annotated in class 2 "intermediary metabolism", there are 2 proteins with only the class 2 annotation and 215 with more detailed annotations, that are thus distributed in the daughter classes: 2.1 – 2.9.

The two teams of biologists that analyzed the above genomes used the results of the bioinformatics methods deployed in AGMIAL, and other available data, to assign a function to the genome proteins. They employed a functional hierarchy that has been previously used to annotate *Bacillus subtilis *[18], called Subtilist hierarchy in the following. This functional hierarchy provides a controlled vocabulary to describe the protein function. Thus they attributed to each protein a node or a leaf of the functional hierarchy. This hierarchy is very simple: it consists of 3 levels that become more specific as one proceeds toward the leaves (see Fig 1).

In this study, we choose to focus only on classes 1, 2 and 3. Class 5 and 6 correspond to proteins for which the annotators judged there was not enough information to conclude on a particular function. Class 4 is a medley that gathers together various heterogeneous functions without any relationship. It was not possible to learn regularities from data of this class. The exclusion of these 3 first level classes and their subclasses in the hierarchy removed 11 out of 62 classes (18%).

#### The descriptors

To generate annotation rules, we have to describe the proteins in terms of their properties. Some properties are intrinsic such as the number of transmembrane segments, the isoelectric point, the molecular mass, the number of domains and their type, etc. Other properties express a relationship between the protein of interest and proteins of other genomes (homology relationship) or between proteins of the analyzed genome (genomic context relationship). These properties are provided by the bioinformatics programs that analyze the genomic data.

#### Homology information

##### • blastmatchGo

For each protein of interest, we use homologous proteins that have been found with BLAST [19]. For the current study we only consider close homologs, i.e., those having more than 50% identical residues and an e-value less than 10^-4^. In addition, the lengths of the protein and its homolog have to be similar to exclude the case of domains (*l*1 ≥ 0.8 × *l*2 or *l*2 ≥ 0.8 × *l*1, with *l*1 the length of the protein and *l*2 the length of its homolog). We then extract the GO-terms [2] associated with the homologous proteins in the Uniprot data bank [20]. The GO-terms correspond to functional classes of the Gene Ontology [21]. A protein has usually many homologs and each homolog can be described by several GO-terms. To build the *blastmatchGo *descriptor we group together all the homologs that have the same GO-term and we consider the fraction (*f*) of homologs that have a particular GO-term.

For instance, this will generate rules such as: '**if ***blastmatchGo*(*esa*100, *GO*: 0006810, *f*) **and ***f *> 0.7 **then **class = 3.5'. In this expression, *esa100 *is the 100th protein of the *L. sakei *genome starting from the origin of replication, *GO:0006810 *is a term of the Gene Ontology that is associated to 70% of the homologs of *esa100 *found by BLAST.

##### • blastmatchSw

The *blastmatchSw *descriptor is similar to the *blastmatchGo *descriptor, but it uses Swiss-Prot (SW) keywords [22] instead of GO-terms to describe homologous proteins.

##### • interpro

This descriptor provides information about domains and motifs. We associate an INTERPRO [23] identifier to a protein if the corresponding domain or motif is found in the protein.

In this study we consider only proteins that have at least one descriptor of *each *type: *blastmatchGo*, *blastmatchSw *and *interpro*. The distribution of these proteins among the nodes of the first level of the Subtilist hierarchy of proteins is shown in Tab. 1 for the two genomes of interest (see also Fig. 1).

**Table 1:** Number of proteins that have at least one descriptor of each type: blastmatchGo, blastmatchSw, interpro.

	Classes			
*Organism*	1	2	3	∑

*L. sakei*	171/367	217/349	229/377	603/1093

*L. bulgaricus*	176/449	190/315	230/381	596/1145

*L. sakei *and*L. bulgaricus *protein distribution at the first level of the functional hierarchy*. a/b*: *a *is the number of proteins with at least one highly similar (percentage of identical residues greater than 50% on a consistent length) protein with a GO-term descriptor, a Swiss-Prot keyword and an INTERPRO domain, *b *is the number of proteins belonging to this class for the considered genome.

#### Intrinsic properties

The descriptors corresponding to intrinsic properties of the proteins considered in this study are:

• **TM **the number of transmembrane segments;

• **pI **the isoelectric point;

• **mm **the molecular weight.

Each protein has many homologs described by GO-terms, SW keywords and INTERPRO identifiers. In order to avoid redundancy and to reduce the search space of the machine learning algorithms, we applied mappings of SW keywords and INTERPRO identifiers to GO-terms. We used the mappings provided on the GO web page [24]. We kept Swiss-Prot keywords and INTERPRO identifiers if no mapping to a GO-term was found. This mapping allows a reduction of the search space that the machine learning algorithm needs to explore.

Table 2 presents the distribution of GO-terms, SW keywords and INTERPRO motifs for the two genomes with and without the application of mappings. We can observe that the size of the search space is significantly reduced (-33% or -40% depending on the genome).

**Table 2:** Impact of the mappings SW → GO and INTERPRO → GO.

*Organism*	GO	SW keywords	INTERPRO	Number of descriptors
*L. sakei*	620/875 (+41,1%)	230/49 (-78,7%)	715/120 (-83,2%)	1565/1044 (-33,3%)

*L. bulgaricus*	599/876 (+46,2%)	223/46 (-80,2%)	1056/199 (-81,1%)	1878/1121 (-40,3%)

*L. sakei *and *L. bulgaricus *numbers of GO-terms, SW keywords and INTERPRO motifs with and without the mapping SW → GO, INTERPRO → GO. *a/b *(*c*%): *a *is the number of different descriptors used without mappings, *b *is the number of different descriptors used with mappings, *c *is the number of descriptors "saved" when carrying out the mapping (in%).

### Two approaches

In this section, we present the two machine learning techniques we used to learn decision-trees: ILP framework and Multilabel probabilistic decision-tree.

#### ILP framework

TILDE is a relational learning system from the ILP community that is based on first-order logical decision-trees. It uses top-down induction of decision-trees by adapting C4.5's heuristics. It allows discretization of numeric attributes and provides look-ahead facilities so that properties of descriptors and parameters can be easily set through a bias file.

We decided to predict protein function by using TILDE level by level, beginning from the upper level of the functional hierarchy. In order to discriminate the three classes of the first level, we build three decision-trees, where each class in turn is considered as the set of examples, while the two others give the counter-examples. Note that with this method a protein may be assigned up to 3 classes of the first level. In order to stay close to the AGMIAL system which allows only one annotation for a protein, we chose to assign a "no prediction" tag to a protein if the three trees disagree on the class predicted. This leads to a decrease in the recall value but, of course, to an increase in the precision.

As the second and third levels contain fewer proteins than the first one, we decided to learn multiclasses trees, that is, trees where each leaf refers to a single class, but where several classes can be found at different leaves. Thus we got ten trees, three at the first level, three at the second level and four at the third level, as only four classes at the second level had subclasses.

#### Multilabel probabilistic decision-tree

In a hierarchical multilabel classification tree, an example may belong to several classes. Moreover, an example belonging to some class with some membership degree also belongs to its superclasses with higher membership degrees.

Each leaf of a probabilistic decision-tree represents a vector of classes where the membership degree is equal to the proportion of the training examples observed in the leaf (and belonging to the class). For example, a leaf may be the vector: (3 – *90%*, 2 – *10%*, 3.2 – *85%*, 3.1 – *15%*, 3.2.3 – *36%*, 3.2.5 – *64%*). Different algorithms, derived from C4.5 [5], have been proposed [25,14]. We chose to use the Clus-HMC algorithm [14] that has been designed to take into account class hierarchy. The algorithm uses minimization of the average variance and a weighted Euclidean distance to compare two partitions of the data. The distance takes into account the depth of the classes in the hierarchy.

In this study, we use the parameters empirically found to be the best by Blockeel *et al. *in [14]. In order to evaluate the methods, we turn to Hierarchical Evaluation Measures, that are adapted to our data.

#### Hierarchical Evaluation Measure

Kiritchenko *et al. *[26] defined a Hierarchical Evaluation Measure which respects the three main properties that a hierarchical evaluation measure should satisfy:

1. The measure gives credit to partially correct classification;

2. The measure punishes distant errors more heavily;

3. The measure punishes errors at higher levels of a hierarchy more heavily.

**Figure 2:**
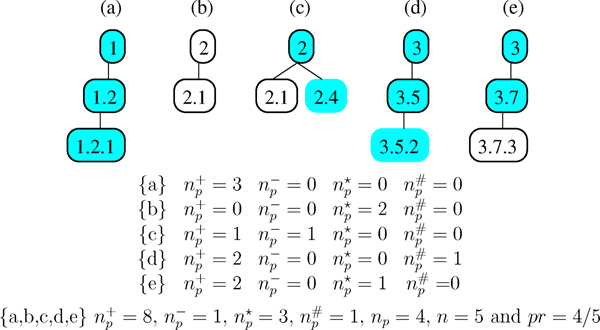
**Hierarchical evaluation measures**. Boxed classes correspond to annotations and filled classes to predictions. *n*_*p*_: number of proteins with at least one prediction (correct or not), $n_{p}^{+}$: number of correct predictions, $n_{p}^{-}$: number of incorrect predictions, $n_{p}^{*}$: number of missing predictions, *n *is the number of proteins to be annotated, *pr *is the fraction of proteins with an annotation.

These properties ensure that we differentiate misclassifications depending on the level at which they occur in the hierarchy. Predictions are evaluated using the five following parameters: *n*: number of proteins to be annotated, *n*_*p*_: number of proteins with at least one prediction (correct or not), $n_{p}^{+}$: number of correct predictions, $n_{p}^{*}$: number of missing predictions, $n_{p}^{\#}$: number of supplementary predictions, and $n_{p}^{-}$: number of incorrect predictions. Fig. 2 illustrates different configurations.

*Hierarchical precision *(*hP*) and *hierarchical recall *(*hR*) have been reformulated with our parameters to respect the three above properties. A *hierarchical Fscore *(*hF*_*β *_∈ [0..1]) has been defined in [26]. The *Fscore *(*hF*_*β*_) measure combines precision (*hP*) and recall (*hR*) to provide a single evaluation of a hierarchical classification tool. This measure is controlled by the *β *∈ [0, + ∞] parameter which permits to give more or less importance to either precision or recall.

Usually, *β *is set to 1 which implies equal weight for precision and recall. These hierarchical measures are defined as follows:

$$\begin{array}{lll} hP & = & \frac{n_{p}^{+}}{n_{p}^{+}+n_{p}^{-}+n_{p}^{\#}}\\ hR & = & \frac{n_{p}^{+}}{n_{p}^{+}+n_{p}^{-}+n_{p}^{*}}\\ hF_{\beta } & = & \frac{(\beta^{2}+1)hP\;hR}{\beta^{2}hP+hR} \end{array}$$

We also employ the prediction rate measure, *pr*, representing the percentage of predicted proteins and defined as *pr *= *n*_*p*_/*n*.

It may happen that some predictions are more detailed than the expert annotation. To respect the spirit of the measures defined in [26], in the evaluation of our method performances we consider the more detailed prediction as an incorrect prediction (see Fig. 2-d). However, a more detailed prediction might very well be correct. Indeed, the annotations considered as references here have been done a couple of years ago with less information than is available today. Consequently, the prediction will often correspond to the annotation that a human expert would do based on the current information available for prediction. For example, in *L. sakei*, protein "DNA directed RNA polymerase, *a *subunit" annotated in class 3.5 (RNA synthesis) is predicted in 3.5.3 (transcription elongation), as it should be.

## Results and Discussion

### Parameters

**Figure 3:**
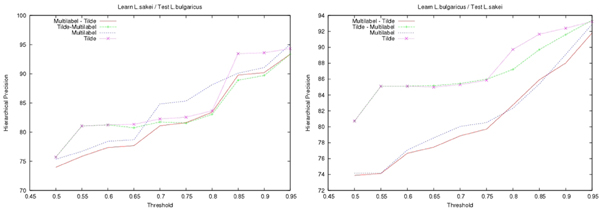
**Hierarchical Precision**. Plot of the hierarchical precision as a function of the confidence threshold (*CT*). A class is predicted only if it represents more than *CT*% of the examples observed in the leaf at the learning stage. On the left hand side *L. sakei *has been used to learn the decision-trees that have then been employed to predict proteins of *L. bulgaricus*, on the right hand side this is the converse.

**Figure 4:**
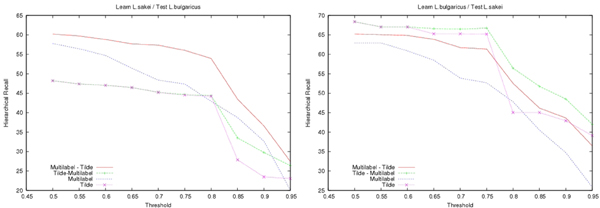
**Hierarchical Recall**. Same as Fig. 3 for the hierarchical recall.

**Figure 5:**
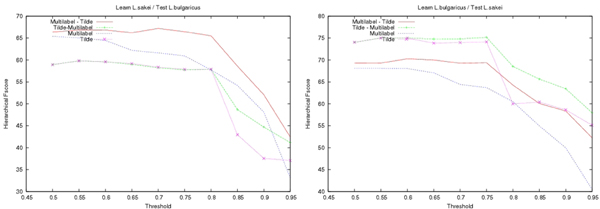
**Hierarchical Fscore**. Same as Fig. 3 for the hierarchical Fscore.

In both approaches, decision-trees were learnt under the same condition: the minimal number of proteins in a leaf had to be equal to 8 (smallest values would likely result in overfitting). When applying decision-tree, a class was predicted only if it represented more than a minimal ratio of the examples observed in the leaf at the learning stage. This minimal ratio called **Confidence Threshold **noted by *CT*, allows us to control the prediction rate. Its value has been chosen empirically. As shown on Fig. 3, the precision increases steadily with the threshold whereas the recall in Fig. 4 exhibits a sharp decrease after this value. As a result, the hierarchical Fscore (*β *= 1) also decreases for thresholds *CT *larger than 75% (see Fig. 5). In the following we use this value *CT *= 75%.

### Approaches

For the two approaches, TILDE and multilabel probabilistic decision tree, we carry out two different tests. In the first test, proteins of both genomes are considered as a whole, and rules are learnt on a fraction of them and tested on the other fraction by a 3-fold cross validation procedure. In the second test, rules are learnt on proteins of a genome and tested on proteins of the second genome. The latter is a more "natural" way of proceeding since we seek to annotate new genomes in the light of previously annotated genomes. Results of these tests are evaluated with the four measures previously presented but we will only detail the second test, which is more natural.

As can be observed in Tab. 3, results are good for both approaches and both genomes. Most of the proteins have a prediction (*pr *> 75% for Multilabel approach and *pr *> 0.96 for TILDE). The recall is in the range 45% and 65% depending on the genome predicted. The precision is good, over 80% for most cases. The resulting hFscore is thus in the range 60% to 70%. Most of the proteins have a good prediction for the first level and some of them have more detailed predictions at the second and third levels.

**Table 3:** Prediction results.

**Learn**	**Test**	**Method**	*hP*	*hR*	*hF*	*pr*
*L. bulgaricus + L. sakei*	3-CV	Multilabel	86.6%	52.2%	65.1%	73.7%
		TILDE	86.7%	51.9%	64.9%	76.4%

*L. sakei*	*L. bulgaricus*	Multilabel	85.3%	47.4%	60.9%	72.2%
		TILDE	82.6%	44.5%	57.8%	96.8%
		combined-Multilabel	81.4%	55.3%	65.9%	98.3%
		combined-TILDE	81.5%	44.7%	57.7%	98.3%

*L. bulgaricus*	*L. sakei*	Multilabel	80.5%	52.7%	63.7%	78.1%
		TILDE	85.9%	65.2%	74.1%	96.8%
		combined-Multilabel	79.7%	61.4%	69.4%	98.5%
		combined-TILDE	86.0%	66.8%	75.2%	98.5%

Results observed for a confidence threshold set to 0.75 in the leaves.

We have also combined the two tested methods as follows:

• Combined-Multilabel: first carry out the prediction with Multilabel. If no prediction is obtained, employ TILDE.

• Combined-TILDE: this is the converse of the previous approach, use TILDE first then Multilabel.

When TILDE is used as the first prediction, no real gain is observed. The prediction rate (*pr*) for the TILDE approach is close to 1 and thus the Multilabel approach is only used for the few proteins that are not predicted by TILDE.

On the other hand, when the Multilabel approach is used as the first prediction method, the gain is important both in terms of recall (almost 10%) and prediction rate (20 to 26%). This increase in the recall is concomitant to a slight decrease in the precision. However, overall, the precision remains close to 0.8 and this is good enough to be used in a semi-automatic application.

### Trees and rules

**Figure 6:**
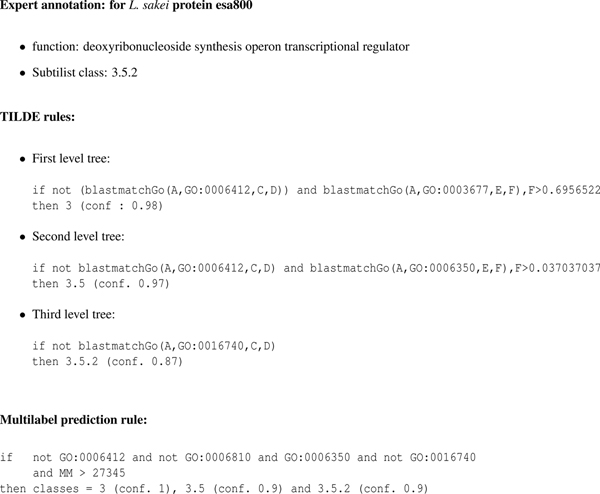
**Example of rules**. Example of rules obtained with TILDE and Multilabel

**Figure 7:**
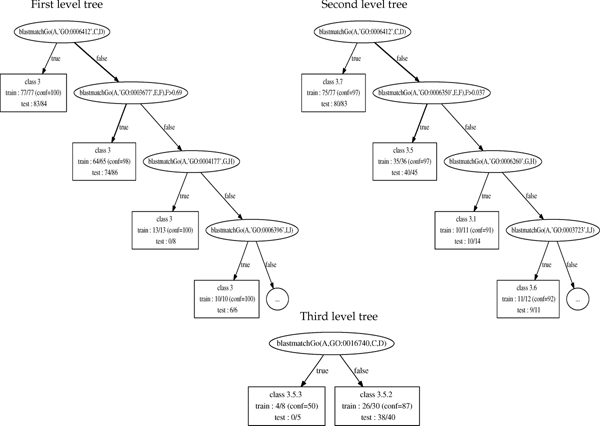
**Example of trees**. Example of trees obtained with TILDE. Note: only the part of the trees corresponding to rules shown in Fig. 6 is displayed.

Fig. 6 presents an example of the rules obtained with TILDE and Multilabel for protein esa800 of *L. sakei*. The trees were learnt with the proteins of *L. bulgaricus*. Fig. 7 shows the trees produced with TILDE at each level (for the first two levels only the fragment of the tree of interest is displayed). The rules correspond to paths in these trees. The meaning of the GO-terms is given in Tab. 4 together with a mapping that most biologists would do of these terms on the functional hierarchy.

**Table 4:** GO-terms.

**GO identifier**	**Definition**	**biologist mapping**
GO:0006412	translation	3.7
GO:0003677	DNA binding	3.1 – 3.5
GO:0004177	aminopeptidase activity	3.10
GO:0006396	RNA processing	3.5 – 3.6
GO:0006350	transcription	3.5
GO:0006260	DNA replication	3.1
GO:0003723	RNA binding	3.6
GO:0016740	transferase activity	context dependent

GO-terms shown in the trees of Fig. 7 and their definition. The last column is the mapping of the GO-term definition on the functional hierarchy that most biologists would do.

Using the tree displayed in Fig. 7, the rules shown in Fig. 6 can be interpreted as follows.

For the first level, the homologs of esa800 do not have the GO-term "translation", but more than 69% of them are associated with the GO term "DNA binding" which is enough to classify the protein in class 3 (conf = 98%) ("information pathways" see Tab. 4).

For the second level, the homologs of esa800 do not have the GO-term "translation" but are associated with the GO-term "transcription" which corresponds to class 3.5 (conf= 97%) (RNA synthesis). For the third level, the homologs of esa800 are not associated with the GO-term "transferase activity". This is a very general term that does not carry any specific information in favor of a particular class. However in the context of class 3.5, it makes sense since the elongation process (3.5.3) corresponds to the attachment (transfer) of a new nucleotide to the growing RNA chain. Therefore the protein is predicted 3.5.2 (conf = 87%) since its homologs do not have this term.

The multilabel approach proposes a similar rule, that concludes to the same class (conf = 90%) for esa800 (Fig. 6).

Annotators can thus easily interpret these rules and trees and confirm or reject the rule conclusion.

## Conclusion and perspectives

Results obtained for the two approaches on both genomes are comparable and are good enough to be useful for the annotators (good precision and high prediction rate). A first attempt at combining the two approaches is very encouraging (this increases the recall and the prediction rate). We will further refine these combinations.

We are now analysing thoroughly the rules obtained from the trees and comparing them in order to extract common pieces of knowledge which could be considered as strongly reliable for an automatic annotation. The biological meaning of these rules and their relevance for annotation purpose will be investigated by experts that use the AGMIAL platform. As we may obtain several possible annotations, we would like to extend the AGMIAL interface in order to make it support multiple annotations for the same protein, if required, and to provide the user with different predictions together with their confidence degree. Also we plan to learn new trees based on a richer set of descriptors for the training examples, for instance, by taking into account the genomic context or subcellular localisation. Finally, we are considering validating our approach by applying it to other genomes and to learn other expressive classifiers.

Note added in proofs: we were considering applying our methodology on the 5 MIPS genomes annotated with the MIPS Funcat functional hierarchy. MIPS scientists published recently a paper [27] describing a work quite similar, in spirit if not in methodological details, to the one we presented here, using Funcat and their 5 annotated genomes.

## Competing interests

The authors declare that they have no competing interests.

## Authors' contributions

JFG, PB, AP and CF designed the research and initiated it. CF and CR suggested to investigate an ILP approache while JA suggested to compare it with an attribute-value based approach. JA and LG performed the research: LG contributed to the development of the approach based on TILDE while JA contributed to that based on the Clus-HMC algorithm. VL prepared the data on the AGMIAL platform. All authors contributed to the analysis of the results, especially CT thanks to her expertise in biology. All authors contributed to the writing of this paper.
